# Optimizing maize yields using growth stimulants under the strategy of replacing chemicals with biological fertilizers

**DOI:** 10.3389/fpls.2022.1069624

**Published:** 2022-11-25

**Authors:** Ahmed I. Abdo, El-Sayed E. A. El-Sobky, Jiaen Zhang

**Affiliations:** ^1^ Department of Ecology, College of Natural Resources and Environment, South China Agricultural University, Guangzhou, China; ^2^ Henry Fok School of Biology and Agriculture, Shaoguan University, Shaoguan, China; ^3^ Soil Science Department, Faculty of Agriculture, Zagazig University, Zagazig, Egypt; ^4^ Agronomy Department, Faculty of Agriculture, Zagazig University, Zagazig, Egypt

**Keywords:** maize, mineral NPK fertilizers, biofertilizers, humic acid, amino acids

## Abstract

Partial replacement of chemicals with biological fertilizers is a recommended strategy to reduce the adverse environmental effects of chemical fertilizer losses. Enhancing the reduced mineral with biological fertilizers strategy by foliar application of humic acid (HA) and amino acids (AA) can reduce environmental hazards, while improving maize (*Zea mays L*.) production under semiarid conditions. The recommended doses of N, P and K (e.g., 286 kg N ha^-1^, 75 kg P_2_O_5_ ha^-1^ and 67 kg K_2_O ha^-1^) were applied as the first fertilization level (100% NPK) and were replaced with biofertilizers by 100%, 75%, 50% and 25% as levels of reducing mineral fertilization. These treatments were applied under four foliar applications of tap water (TW), HA, AA and a mixture of HA and AA. Our results reported significant reductions in all parameters, including maize ear yield attributes and grain nutrient uptake, when replacing the mineral NPK with biofertilizers by 25-100% replacement. However, these reductions were mitigated significantly under the application of growth stimulants in the descending order: HA and AA mixture>AA>HA>TA. Applying a mixture of HA and AA with 75% NPK + biofertilizers increased ear length, grain yield, grain uptake of N and K, and crude protein yield by 37, 3, 4, 11 and 7%, respectively as compared with 100% mineral fertilizer only. Moreover, all investigated parameters were maximized under the application of 75% NPK + biofertilizers combined with AA or the mixture of HA and AA, which reveals the importance of growth stimulants in enhancing the reduced chemical NPK strategy. It could be concluded that the mineral NPK rate can be reduced by 25% with biofertilization without any yield losses when combined with HA and AA under arid and semi-arid conditions. That achieves the dual goals of sustainable agriculture by improving yield, while reducing environmental adverse effects.

## 1 Introduction

Maize (*Zea mays L*.) is the most important staple crop worldwide with various basic uses, such as human diets, animal feeding and energy production. The global area of maize production was greater than 150×10^6^ ha in 2010 (Bassu et al., 2014), and the demand is expected to double by 2050 (Ramirez-Cabral et al., 2017). In Egypt, maize is the second main crop (7.5×10^6^ tons) with an area of 1.1×10^6^ ha that is located in a semiarid region with low-fertility soil (FAO, 2020). On average, 290, 80 and 70 kg ha^-1^ of N, P_2_O_5_ and K_2_O, respectively, are the conventional mineral fertilization to maize fields in Egypt with use efficiencies by 30, 36 and 20%, respectively (El-Etr and Mahmoud, 2011; El-Gedwy, 2020; El-Sobky and Abdo, 2020). This means that more than 60% of the applied synthetic fertilizers are lost to the environment, which causes environmental hazards and economic losses. Furthermore, intensive nitrogen fertilization can decrease crop yields owing to lodging (Corbin et al., 2016) in addition to inducing water and air pollution as a result of N losses (Huang et al., 2017) through nitrate leaching (Fan et al., 2012) and nitrous oxide and ammonia emissions (Hirel et al., 2011).

For cleaner production, intensive research work has been carried out to increase nutrient use efficiencies in parallel with reducing synthetic fertilizer usage and losses. Biofertilizers have been suggested as inputs for sustainable agricultural production, as they are eco-friendly and cost-effective materials (Kumawat, 2017). Biofertilizers are defined as the formulations containing living microorganisms or latent cells having the potential of colonizing roots of crops plants and promoting the growth by improving nutrients availability and acquisition (Lakshmi, 2014; du Jardin, 2015).

Chemical fertilizers provide root zone with readily available nutrients that are subject to losses, while biofertilizers increase nutrient uptake by fixing the nutrients that are vulnerable to loss and from outer sources (e.g., N_2_ fixing bacteria) or by solubilizing unavailable nutrients (e.g., P and K solubilizing bacteria) (Pawar et al., 2019). Biofertilizers are sources of beneficial soil microorganisms, which enhance plant growth, yield and N use efficiency by increasing the availability and supply of essential nutrients (Kubheka et al., 2020a; Phares et al., 2022). Also, Biofertilizers improve plant resistance to environmental stress, including drought, temperature and saline conditions (Itelima et al., 2018). Maize yields were optimized under the reduced fertilization strategy when combined with N, P and K biofertilizers (Jilani et al., 2007; Yosefi et al., 2011). On the other hand, using biofertilizers improved maize yields by only 15.3% on average in a meta-analysis study (Schmidt and Gaudin, 2018). We hypothesized that applying growth stimulants, such as humic and amino acids, can enhance maize growth and yields under a reduced synthetic N strategy with biofertilizers.

Humic acid improves the morphological and yield attributes; metabolism (e.g., total soluble sugar, photosynthetic pigment, total carbohydrates, proline and total amino acids); nutrient contents, nutrients uptake and yields and yield attributes (Canellas et al., 2019; Khan et al., 2019; Yuan et al., 2022). Amino acids enhance plant functions such as photosynthesis, protein synthesis, phytohormone activators, stoma action, stress resistance and chelating effects (Matysiak et al., 2020). Amino acids are better than humic acid in improving the maize yield attributes and grain contents of N, P and K and have positive effects on the physicochemical processes and yield attributes (Ragheb, 2016). Amino acids are readily available sources of N, protein synthesis, and hormone precursors, including auxins and antistress agents, which in turn positively affect plant growth and yields. However, there was no documentation in the literature on the effects of the combined foliar application of humic and amino acids with partial replacement of NPK mineral fertilizers with biofertilizers on maize yield quantities and crop grain qualities.

Therefore, this study aimed to evaluate the possibility of reducing nutrient surplus by growth stimulants (amino or humic acid) to enhance maize yield attributes and nutrients uptake under replacement of synthetic fertilizers strategy with biofertilizers. This study also aimed to select the best combined rate of minerals and biofertilizers when using single or mixed humic and amino acids for optimal maize production under semiarid conditions. This study serves the efforts of achieving the dual goals of sustainable agriculture by maintaining optimal yields accompanied by less environmental effects, especially in arid and semi-arid regions.

## 2 Materials and methods

### 2.1 Experiment site

A two-season trial was carried out at the Experimental Station in Ghazala Village, Fac. of Agric., Zagazig Univ., Sharkia Governorate, Egypt (30.11°N, 31.41°E) during the summer seasons of 2019 and 2020. This site is described by hot weather, dry summer seasons (**Table S1**) with an average temperature of 32.1°C and no precipitation. Analysis of soil was carried according to  Klute (1986), the soil is alluvial clay in texture (FAO-UNESCO soil map) and consisted of 475.7 ± 2.2 and 476.6 ± 1.8 g kg^-1^ clay, 318.2 ± 0.8 and 318 ± 1.1 g kg^-1^ silt and 206.1 ± 1.2 and 205.4 ± 1.3 g kg^-1^ sand during the first and second seasons, respectively. The soil pH levels (1:2.5) were 8.05 ± 0.02 and 8.02 ± 0.05, respectively and the EC values (1:5) (dSm^-1^) were 1.85 ± 0.1 and 1.92 ± 0.06 during the first and second seasons, respectively. The available N, P and K (mg kg^-1^) concentrations were 21.12 ± 1.1 and 22.15 ± 0.9, 8.15 ± 0.9 and 8.22 ± 0.8, and 149.3 ± 1.5 and 148.7 ± 1.3 during the first and second seasons, respectively. The soil organic carbon contents were 7.45 ± 0.13 and 7.56 ± 0.04 g kg^-1^, respectively.

### 2.2 Experimental design and study factors

In total, twenty treatments with three replicates were conducted in a randomized complete block split-plot design. Foliar spraying using growth stimulants was used for the main plot, and mineral and biofertilizer applications were used for the subplots. Three foliar sprays with humic acid (HA), amino acids (AA) and a mixture of HA+AA were applied at rates of 3 g L^-1^, 3 ml L^-1^ and 3 g L^-1^+3 ml L^-1^, respectively. In parallel, tap water (TW) was sprayed as the control. Foliage-applied treatments were carried out using water (595 L ha^-1^ per spray) at 21, 35 and 55 days after planting (DAP). The foliar spraying of humic acid and amino acids was conducted by using solid and liquid commercial products, namely, K-humate (e.g., 860 g kg^-1^ humic acid, total organic matter 750 g kg^-1^, pH 5.5–6.5 and 12 g kg^-1^ K_2_O), as well as Aminocat star (Shoura, Alexandria, Egypt) as a source of amino acids containing 10 g kg^-1^ free amino acids, 3 g kg^-1^ N, 1 g kg^-1^ P_2_O5 and 5 g kg^-1^ K_2_O.

Five rates of mineral and biofertilizer application (e.g., NPK 100% (F1), NPK 75% plus biofertilizers (F2), NPK 50% plus biofertilizers (F3), NPK 25% plus biofertilizers (F4) and biofertilizers (F5)) were applied. The recommended doses of NPK (NPK 100%) were established by adding 286 kg N ha^-1^ ammonium nitrate (335 g kg^-1^ N), 75 kg P_2_O_5_ ha^-1^ superphosphate (155 g kg^-1^ P_2_O_5_) and 67 kg K_2_O ha^-1^ potassium sulfate (48 g kg^-1^ K_2_O). The recommended NPK doses are applied by maize producers for commercial production in the region. Before planting, the maize seeds were inoculated with a biofertilizer mixture (e.g., Nitrobein biofertilizer containing *Azotobacter* sp. and *Azospirillum* sp. as N_2_-fixing bacteria, phosphorine biofertilizer containing *Bacillus megaterium* var. *phosphaticum* as phosphate-solubilizing bacteria, and potassiomage as K solubilizing bacteria). These biofertilizers were produced by the Agriculture Research Center, Giza, Egypt and were used at the recommended dose of 1 kg ha^-1^ for each biofertilizer. Superphosphate and potassium sulfate were applied basally before planting. Nitrogen fertilizer was applied in two equal splits before the first and second irrigation periods at 21 and 34 days after planting (DAP).

### 2.3 General agronomic practices

During the two seasons, maize was cultivated after wheat (*Triticumaestivum L.*) and the soil was plowed using a moldboard plow to a depth of 0.30 m and was divided into 60 plots. The area of each plot was 3.5 m x 5 m including 5 ridges with 70 cm apart. On May 15^th^ and 20^th^ of the first and second seasons, a single cross 178 yellow maize cultivar was planted. Seeds were sown by hand at a rate of 24 kg ha^-1^ in both seasons on one side of the ridge in hills that were 25 cm apart. Furrow flood irrigation was conducted at each 14-day interval with a total amount of 7140 m^3^ ha^-1^. The plants were thinned before the first irrigation (21 DAP) to one plant for each hill to a density of 57120 plants ha^-1^. Soil samples were collected each season before planting at a depth of 0-30 cm to determine the soil physical and chemical properties.

### 2.4 Recorded data

#### 2.4.1 Maize yields and yield attribute measurements

By late September of each year, the maize was harvested (120 DAP), and the following yield attributes were recorded using ten ears: ear length (cm), ear diameter, row number per ear, grain number per row, grain number/ear (calculated), 100-grain weight (g), and grain weight per ear (g). Additionally, the following final yield traits were recorded from the three central ridges at each plot and were converted into Mg ha^-1^: grain yield at a grain moisture content of 15.5%, ear yield, stover yield and biological yield. The harvest index was calculated from the grain and total yields (Mg ha^-1^) according to (Buresh et al., 1988) as follows:


$$\text{Harvest index }\left(\text{HI}\right)=\frac{\text{grain yield }}{\text{total yield}}\times 100$$

#### 2.4.2 Determination of macronutrients content and uptake

The grain samples were dried at 70°C after harvest to determine their total N, P and K contents according to (Faithfull, 2002). The grain N, P and K uptakes (kg ha^-1^) were calculated by multiplying the grain yields by the grain N, P and K percentages (Moll et al., 1962). The grain protein contents (%) were calculated by multiplying the grain N percentages by 5.70 (Bishnoi and Hughes, 1979). The crude protein yields (CPY) (kg ha^-1^) were calculated by multiplying the grain yields (kg ha^-1^) by the percentages of grain protein content (%).

### 2.5 Statistical analysis

The data were statistically analyzed using MSTAT-C Version 2.1, which was used also for analysis of variance (ANOVA) determinations (Gomez and Gomez, 1984). The treatment means were compared using the least significant differences (LSD) test at a 0.05 probability level (Snedecor and Cochran, 1989). The Pearson’s simple correlation matrix for yields, yield attributes and uptake of nutrients in grains was also computed by SPSS 20. The path coefficient analysis was estimated. Path-coefficient analysis measures the direct effect of one predictor variable on another and has been widely used to determine the nature of the relationships among grain yields and their contributing components (Pavlov et al., 2015).

## 3 Results

### 3.1 Maize yield attributes and crude protein yield

The greatest ear length (EL) (20.32 cm) was reported under the application of 75% NPK + biofertilizers with HA and AA mixture during the first season, while the greatest ear length was (20.50 cm) during the second season under the application of 100% NPK and AA without biofertilizers (**Table 1**). On average, ELs exhibited their maximum (e.g., 17.99 and 18.83 cm) values under the application of 75% NPK + biofertilizers when compared with all other NPK and biofertilizer combinations during the first and second seasons, respectively. Additionally, ELs exhibited their maximum lengths (e.g., 17.74 and 17.71 cm) under HA and AA mixture when compared with the control [e.g., tap water (TW)] and the single application of HA or AA during the first and second seasons, respectively. Similarly, ear diameter (ED), number of grains per ear (NG/E) and grain weight per ear (GW/E) were maximized under the application of 75% NPK + biofertilizers and HA and AA mixture as compared with the other single applications of growth stimulants during both seasons (**Tables 1**, **2**). The NG/Es had the highest values (e.g., 614.6 and 589.9) under the application of 50% NPK + biofertilizers with HA and AA mixture or 100% NPK and AA, respectively. In contrast, the EDs (cm) and GW/Es (g) exhibited their highest values (e.g., 4.38 and 4.48, 229.5 and 225.9, respectively) under the combined application of 75% NPK + biofertilizers and an HA and AA mixture during the first and second seasons, respectively. On the other hand, the application of 100% NPK with TW resulted in the highest 100-grain weights (e.g., 37.91 and 40.51 g) during the two seasons. On average, the 100-grain weights were maximized under the application of 75% NPK + biofertilizers with foliar application of the HA and AA mixture during the two seasons. The application of growth stimulants (HA and/or AA) significantly improved all ear parameters compared with TW, and the mixture exhibited the highest values. Application of these stimulants reduced the negative impact of replacing mineral fertilizer with biofertilizers on the ear parameters, while using only 25% NPK with biofertilizers under the application of an HA and AA mixture exhibited all investigated ear parameters to be higher, equal or have no significant reductions when compared with using 100% mineral fertilizer.

**Table 1:** Impact of foliar spraying of stimulants and chemical and bio fertilization treatments on ear length, ear diameter and number of grains per ear of maize.

Foliar spraying	EL						ED						NG/E					
	F1	F2	F3	F4	F5	Mean	F1	F2	F3	F4	F5	Mean	F1	F2	F3	F4	F5	Mean
2019 season																		
TW	14.88g	18.25bc	17.50cd	16.69de	15.57e:g	*16.58^c^*	4.35a	3.98f:h	3.75ij	3.75ij	4.10c:f	*3.99^c^*	543.3cd	457.4hi	450.8i	465.9g:i	490.9f:h	*481.6^d^*
HA	17.25cd	16.63de	15.82e:g	17.25cd	15.25fg	*16.44^c^*	4.17b:e	4.27a:c	4.03e:g	4.07d:g	3.82hi	*4.07^b^*	533.6c:e	554.9c	457.4hi	490.9f:h	516.8d:f	*510.7^c^*
AA	19.50ab	16.75de	16.50d:f	16.63de	16.50d:f	*17.18^b^*	4.09d:f	4.33ab	4.08d:g	4.23a:d	3.60j	*4.06^b^*	537.1c:e	606.6ab	505.2ef	559.1c	466.7g:i	*534.9^b^*
HA+AA	16.75de	20.32a	15.63e:g	19.25ab	16.75de	*17.74^a^*	3.92g:i	4.38a	4.27a:c	4.32ab	4.05e:g	*4.18^a^*	500.1e:g	569.2bc	614.6a	558.1c	613.1a	*571.0^a^*
Mean	17.09^B^	17.99^A^	16.36^C^	17.45^AB^	16.02^C^		4.13^B^	4.24^A^	4.03^C^	4.09^BC^	3.89^D^		528.5^AB^	547.0^A^	507.0^C^	518.5^BC^	521.9^BC^	
2020 season																		
TW	14.63j	19.25ab	16.50e:h	17.57de	17.19ef	*17.03^b^*	4.45ab	4.03e:g	3.55j	3.85g:i	4.00f:h	*3.98^c^*	546.0c:f	519.0f:h	475.0i	494.8hi	525.6e:h	*512.1^c^*
HA	17.75c:e	18.88bc	15.94f:i	16.75e:h	14.75ij	*16.81^b^*	4.29bc	4.29bc	4.18c:e	3.99f:h	3.84hi	*4.12^ab^*	525.6e:h	563.4a:c	496.3g:i	497.0g:i	508.2gh	*518.1^c^*
AA	20.50a	17.25e	17.50de	16.88e:g	15.50h:j	*17.53^a^*	4.07d:f	4.48a	4.03e:g	4.18c:e	3.70ij	*4.09^b^*	589.9a	558.8a:d	554.2b:e	528.4d:g	439.3j	*534.1^b^*
HA+AA	16.75e:h	19.94ab	15.88g:j	18.75b:d	17.25e	*17.71^a^*	4.04ef	4.23cd	4.29bc	4.14c:f	4.15c:f	*4.17^a^*	526.3d:h	567.0a:c	565.9a:c	583.4ab	577.2a:c	*563.9^a^*
Mean	17.41^B^	18.83^A^	16.45^C^	17.49^B^	16.17^C^		4.21^A^	4.25^A^	4.01^BC^	4.04^B^	3.92^C^		546.9^A^	552.1^A^	522.8^B^	525.9^B^	512.5^B^	

TW, tap water, HA, humic acid, AA, amino acids, HA + AA, mixture of humic acid + amino acids, F1, 100% NPK, F2, 75% NPK + biofertilizers, F3, 50% NPK + biofertilizers, F4, 25% NPK + biofertilizers, F5, biofertilizers, EL is ear length (cm), ED is ear diameter (cm) and NG/E is number of grains/ear. Means in italic refer to foliage applications, while none italic refer to fertilization treatments. Means followed by different letters in the same direction differ significantly by LSD (p ≤ 0.05).

**Table 2:** Impact of foliar spraying of stimulants and chemical and bio fertilization treatments on grain weight per ear,100-grain weight and grain yield of maize.

Foliar spraying	GW/E						100-GW											GY												
	F1	F2	F3	F4	F5	Mean	F1	F2		F3		F4		F5		Mean		F1			F2		F3		F4		F5	Mean		
2019 season																														
TW	206.0b	122.6ij	111.0j	109.9j	150.0f:h	*139.9^d^*	37.91ab		26.81i:k		24.65jk		23.58k		30.55e:i		*28.70^b^*		8.79a:c		5.38g:i		4.88h		4.83h		5.64e:h			*5.90^c^*
HA	185.0c	175.9cd	156.1e:g	150.8f:h	125.2ij	*158.6^c^*	34.66b:d		31.69c:g		34.14b:e		30.72d:h		24.20jk		*31.08^a^*		6.91c:g		7.73b:d		6.36d:h		6.63d:h		4.95h			*6.51^bc^*
AA	133.5hi	213.6ab	172.3c:e	182.7c	139.5g:i	*168.3^b^*	24.86jk		35.23bc		34.10b:e		32.72c:f		29.90f:i		*31.36^a^*		7.33c:g		9.39ab		7.57b:e		8.02b:d		5.63f:h			*7.59^ab^*
HA+AA	159.3d:f	229.5a	171.5c:e	174.8cd	169.9c:e	*181.0^a^*	31.84c:g		40.31a		28.03g:j		31.30d:h		27.70h:j		*31.83^a^*		7.00c:g		10.08a		7.54b:f		7.18c:g		6.96c:g			*7.75^a^*
Mean	170.9^B^	185.4^A^	152.7^C^	154.5^C^	146.1^C^		32.32^A^		33.51^A^		30.23^B^		29.58^BC^		28.09^C^				7.51^AB^		8.15^A^		6.59^BC^		6.67^BC^		5.80^C^			
2020 season																														
TW	221.2ab	118.0jk	106.8k	104.7k	130.2ij	*136.2^d^*	40.51a		22.74j		22.49j		21.16j		24.82h:j		*26.34^c^*		9.72ab	5.20jk		4.70k		4.60k		5.73ij			*5.99^d^*	
HA	181.7de	183.0de	149.6gh	145.6g:i	114.1jk	*154.8^c^*	34.57bc		32.51c:e		30.19d:g		29.30e:g		22.44j		*29.80^b^*		7.98de	8.04de		6.57gh		6.41g:i		5.01jk			*6.80^c^*	
AA	137.7hi	225.9a	155.4f:h	169.7ef	137.5hi	*165.2^b^*	23.34ij		40.43a		28.05f:h		32.11c:e		31.38c:f		*31.06^a^*		6.05hi	9.93a		6.82f:h		7.46ef		6.05hi			*7.26^b^*	
HA+AA	155.4f:h	206.1bc	189.4cd	190.0cd	155.9fg	*179.3^a^*	29.56d:g		36.34b		33.49b:d		32.58b:e		27.01g:i		*31.79^a^*		6.82f:h	9.06bc		8.32cd		8.36cd		6.86fg			*7.88^a^*	
Mean	174.0^A^	183.3^A^	150.3^B^	152.5^B^	134.4^C^		32.00^A^		33.00^A^		28.55^B^		28.79^B^		26.41^C^				7.64^A^	8.06^A^		6.60^B^		6.70^B^		5.91^C^				

TW, tap water, HA, humic acid, AA, amino acids, HA + AA, mixture of humic acid + amino acids, F1, 100% NPK, F2, 75% NPK + biofertilizers, F3, 50% NPK + biofertilizers, F4, 25% NPK + biofertilizers, F5, biofertilizers, GW/E is grain weight/ear (g), 100-GW is 100- grain weight (g) and GY is grain yield (Mg ha^-1^). Means in italic refer to foliage applications, while none italic refer to fertilization treatments. Means followed by different letters in the same direction differ significantly by LSD (p ≤ 0.05).

The grain yield (GY), stover yield (SY), biological yield (BY), harvest index (HI) and crude protein yield (CPY) responded differently to the combinations of minerals and biofertilizers and growth stimulants (**Tables 2**–**4**). The GYs and EYs (Mg ha^-1^) exhibited their highest values (e.g., 10.03 and 9.93, 11.49 and 11.63, respectively) when applying 75% NPK + biofertilizers combined with the HA and AA mixture and the single application of AA during the first and second seasons, respectively. On average, the GYs and EYs recorded their maximum values under the application of 75% NPK + biofertilizers with foliar application of the HA and AA mixture during the two seasons. The maximum values of SY (Mg ha^-1^) and BY (Mg ha^-1^) during the first and second seasons (e.g., 21.72 and 20.19 and 32.61 and 30.60, respectively) were obtained when applying 75% NPK + biofertilizer treatment combined with AA during the first season and the mixture of HA and AA during the second season, respectively. In contrast, the HIs (%) reached their highest values (e.g., 45.60 and 45.42) under the application of AA combined with 25% NPK + biofertilizers in the first season and biofertilizers without mineral fertilization in the second season. Applying the 75% NPK + biofertilizer treatment combined with the application of HA and AA reported the highest improvements in GY, EY, SY and BY, which was followed by 100% NPK, with no significant differences. In contrast, the biofertilization treatment recorded the highest HI, while the lowest HI was exhibited under mineral fertilization (100% NPK) only. GY, SY and BY were sensitive to the replacement of mineral NPK with biofertilizers under TW, with average reductions of 44.44, 33.26 and 38.41%, respectively, when compared with mineral fertilizer with biofertilization. The maximum values of CPY (kg ha^-1^) during the first and second seasons (e.g., 1064.2 and 1089, respectively) were reported under the 75% NPK + biofertilizer treatment combined with application of the HA and AA mixture during the first season and AA during the second season.

**Table 3:** Impact of foliar spraying of stimulants and chemical and bio fertilization treatments on stover, ear and biological yields of maize.

Foliar spraying	SY						EY										BY																		
	F1	F2	F3	F4	F5	Mean	F1	F2		F3		F4		F5		Mean	F1				F2			F3			F4			F5			Mean		
2019 season																																			
TW	13.57de	8.79f:j	9.64f:h	9.43f:i	5.06k	*9.30^c^*	10.47b		6.16ij		5.63j		5.84j		7.61fg		*7.14^d^*		24.04c			14.95g:i			15.27f:h			15.26f:h			12.67i				*16.44^c^*
HA	17.63bc	15.76:d	9.12f:i	7.06h:k	6.58i:k	*11.23^bc^*	9.47c		8.97c		7.86ef		7.72f		6.44h:j		*8.09^c^*		27.10b			24.72bc			16.98e:g			14.77g:i			13.02hi				*19.32^b^*
AA	11.20ef	21.72a	9.68f:h	8.34g:j	5.93jk	*11.37^b^*	6.81g:i		10.89ab		8.80cd		9.26c		7.16f:h		*8.58^b^*		18.01ef			32.61a			18.48de			17.60e:g			13.08hi				*19.96^b^*
HA+AA	13.10de	19.42ab	18.00bc	10.70e:g	8.72f:j	*13.99^a^*	7.96d:f		11.49a		8.71c:e		8.90cd		8.71c:e		*9.15^a^*		21.06d			30.91a			26.71bc			19.59de			17.42e:g				*23.14^a^*
Mean	13.87^B^	16.42^A^	11.61^C^	8.88^D^	6.57^E^		8.67^B^		9.37^A^		7.75^C^		7.93^C^		7.48^C^				22.55^B^			25.80^A^			19.36^C^			16.81^D^			14.05^E^				
2020 season																																			
TW	12.00d:f	8.96f:i	10.63e:g	8.48g:i	6.89hi	*9.39^d^*	11.40a		5.96ij		5.47j		5.56j		6.76hj			*7.03^d^*		23.40d			14.90i:l			16.10g:k			14.04kl			13.65kl		*16.42^d^*	
HA	9.21fgh	14.72cd	10.15e:g	8.42g:i	8.77g:i	*10.25^c^*	9.44cd		9.28cd		7.63e:h		7.40f:h		5.89ij			*7.93^c^*		18.65e:g			23.99cd			17.78e:h			15.81h:l			14.65j:l		*18.18^c^*	
AA	16.96bc	16.83bc	12.12de	8.85g:i	6.30i	*12.21^b^*	7.00gh		11.63a		7.87e:g		8.54de		7.09f:h			*8.42^b^*		23.96cd			28.46ab			19.99e			17.39e:i			13.39l		*20.64^b^*	
HA+AA	19.33ab	20.19a	16.43bc	9.88e:g	9.14f:i	*14.99^a^*	7.86e:g		10.42b		9.65bc		9.72bc		7.92ef			*9.11^a^*		27.19b			30.60a			26.08bc			19.60ef			17.06f:j		*24.10^a^*	
Mean	14.37^A^	15.17^A^	12.33^B^	8.90^C^	7.77^C^		8.92^A^		9.32^A^		7.65^B^		7.80^B^		6.91^C^					23.30^A^			24.49^A^			19.98^B^			16.71^C^			14.69^D^			

TW, tap water, HA, humic acid, AA, amino acids, HA + AA, mixture of humic acid + amino acids, F1, 100% NPK, F2, 75% NPK + biofertilizers, F3, 50% NPK + biofertilizers, F4, 25% NPK + biofertilizers, F5, biofertilizers, SY is stover yield (Mg ha^-1^), EY is ear yield (Mg ha^-1^) and BY is biological yield (Mg ha^-1^). Means in italic refer to foliage applications, while none italic refer to fertilization treatments. Means followed by different letters in the same direction differ significantly by LSD (p ≤ 0.05).

**Table 4:** Impact of foliar spraying of stimulants and chemical and bio fertilization treatments on harvest index and crude protein yield of maize.

Foliar spraying	HI						CPY					
	F1	F2	F3	F4	F5	Mean	F1	F2	F3	F4	F5	Mean
2019 season												
TW	36.54a:e	36.01a:e	32.01c:e	31.69c:e	45.40a	*36.33^ab^*	884.1a:c	643.8e:h	487.9f:i	416.2i	482.4f:i	*582.9^b^*
HA	25.81e	31.28c:e	37.60a:e	44.97ab	38.19a:e	*35.57^ab^*	754.2c:e	881.6a:c	452.2g:i	697.8c:f	457.1g:i	*648.6^b^*
AA	40.53a:d	28.86de	40.96a:d	45.60a	43.23a:c	*39.83^a^*	878.2a:d	1013.7ab	776.6cde	820.8b:e	436.8hi	*785.2^a^*
HA+AA	33.31a:e	32.61b:e	28.25de	36.65a:e	39.95a:d	*34.15^b^*	818.9b:e	1064.2a	662.8d:g	787.1cde	676.7c:f	*801.9^a^*
Mean	34.05^BC^	32.19^C^	34.70^BC^	39.73^AB^	41.69^A^		833.8^A^	900.8^A^	594.9^BC^	680.5^B^	513.2^C^	
2020 season												
TW	41.52a:c	34.83c:g	29.18gh	32.77fg	42.01ab	*36.06^ab^*	1000.0ab	636.2gh	412.2k	463.4jk	493.2jk	*601.0^d^*
HA	42.85ab	33.52e:g	37.01b:f	40.58a:d	34.28d:g	*37.65^a^*	884.0cd	942.9bc	705.1f:h	515.4ij	423.9jk	*694.2^c^*
AA	25.37h	34.98c:g	34.68d:g	42.86ab	45.42a	*36.66^a^*	757.7ef	1089.0a	705.7fg	777.6ef	476.5jk	*761.3^b^*
HA+AA	25.16h	29.59gh	31.94f:h	42.66ab	40.18a:e	*33.91^b^*	820.1de	961.4bc	927.6bc	747.1ef	610.1hi	*813.3^a^*
Mean	33.72^B^	33.23^B^	33.20^B^	39.72^A^	40.47^A^		865.5^A^	907.4^A^	687.7^B^	625.9^C^	500.9^D^	

TW, tap water, HA, humic acid, AA, amino acids, HA + AA, mixture of humic acid + amino acids, F1, 100% NPK, F2, 75% NPK + biofertilizers, F3, 50% NPK + biofertilizers, F4, 25% NPK + biofertilizers, F5, biofertilizers, HI is harvest index (%) and CPY is crude protein yield (kgha^-1^). Means in italic refer to foliage applications, while none italic refer to fertilization treatments. Means followed by different letters in the same direction differ significantly by LSD (p ≤ 0.05).

### 3.2 Macronutrient content and uptake

**Figure 1:**
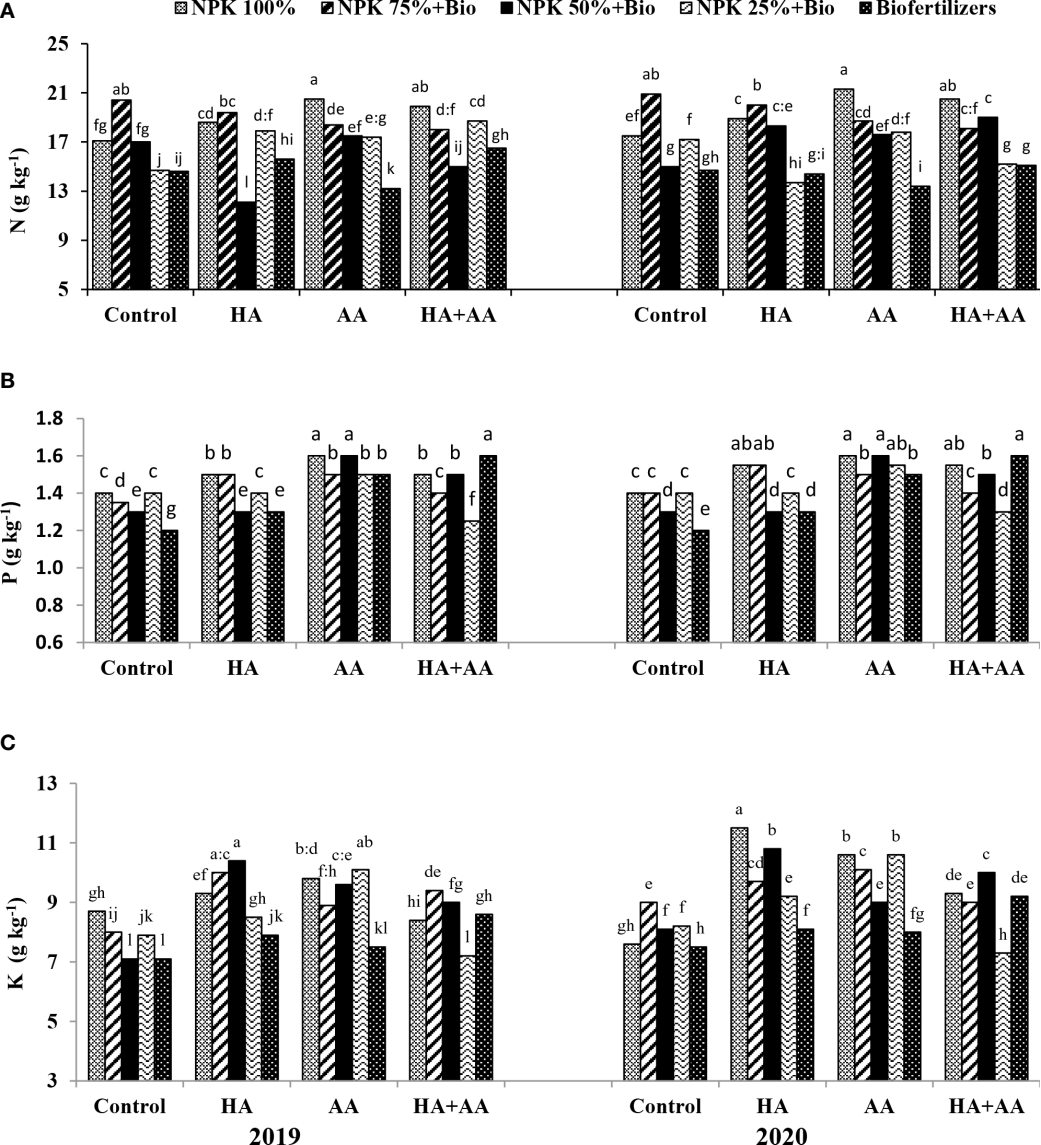
Impact of humic acid (HA), amino acids (AA) and the mixture (HA+AA) application on contents of nitrogen (%) **(A)**, phosphorous (%) **(B)** and potassium (%) **(C)** under chemical and bio fertilization treatments. Letters above columns refer to the significance LSD (p ≤ 0.05).

**Figure 2:**
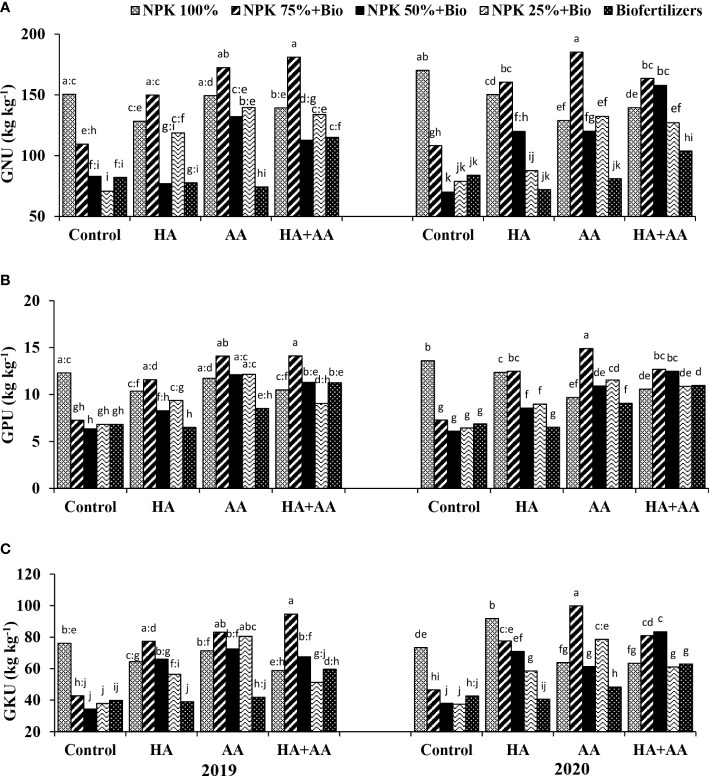
Impact of humic acid (HA), amino acids (AA) and the mixture (HA+AA) application on grain nitrogen uptake (GNU) **(A)**, grain phosphorus uptake (GPU) **(B)** and grain potassium uptake (GKU) **(C)** under five fertilization treatments. Letters above columns refer to the significance LSD (p ≤ 0.05).

The application of 100% NPK with AA or 75% NPK + biofertilizer treatments with TW resulted in the highest N contents (e.g., 20.5 and 20.4 g kg^-1^) during the 1^st^ season and 21.3 and 20.9 g kg^-1^ during the 2^nd^ season, respectively (**Figure 1A**). The N content decreased significantly with replacing the mineral NPK by more than 50%, where the N content decreased from 19.5 g kg^-1^ under 100% NPK with AA or 75% NPK + biofertilizer treatments to 14.5 g kg^-1^ under biofertilization only. The maximum P content (1.6 g kg^-1^) was reported under the application of 100% NPK and 50% NPK + biofertilizers combined with AA or the application of biofertilizers combined with HA and AA mixture during both seasons (**Figure 1B**). On average, the P content was the highest (1.54 g kg^-1^) when applying 100% NPK and decreased significantly with replacing the mineral fertilization until reaching 1.4 g kg^-1^ under the biofertilization treatment. The AA was higher than HA and AA mixture followed by HA and finally TW for their effects on increasing P contents. The maximum K contents (10.4 and 10.8 g kg^-1^) during the 1^st^ and 2^nd^ seasons, respectively, were exhibited with the application of 50% NPK + biofertilizers with HA (**Figure 1C**). The HA or AA resulted in the highest K contents (e.g., 9.2 and 9.7 g kg^-1^, respectively), while TW exhibited the lowest K contents (e.g., 7.7 and 8.0 g kg^-1^) during the 1^st^ and 2^nd^ seasons, respectively. The grain uptakes of N (GNU), P (GPU) and K (GKU) improved significantly in response to the application of growth stimulants, even with reductions in the mineral NPK application rates (**Figure 2**). GNU (kg kg^-1^), GPU (kg kg^-1^), GKU (kg kg^-1^) and CPY (kg ha^-1^) exhibited their maximum values (e.g., 181, 14.12 and 94.36, respectively) when 75% NPK + biofertilizers were applied combined with foliar application of the HA and AA mixture during the first season. While applying 75% NPK + biofertilizers combined with foliar application of AA maximized these parameters (e.g., 185.2, 14.9 and 99.75, respectively) during the 2^nd^ season. Generally, a reduction by 47% was reported in these parameters under partial or complete replacement of mineral NPK without growth stimulants. However, the application of HA with AA or a single AA mitigated this reduction significantly, especially under 75% or 50% NPK with biofertilizer treatments.

### 3.3 Correlations and path coefficients among the studied variables

The EL was significantly and positively correlated with GN/E, stover and biological yields, crude protein yield, N (%) and GNU when the data were pooled over the two years (**Table 5**). Additionally, NG/E had positive and significant correlations with yield attributes, CPY, and macronutrient contents and uptake. The 100-grain weight exhibited positive and significant correlations with SY, EY, BY, CPY, K (%), GNU, GPU, GKU and GY. Moreover, SY was positively and significantly correlated (*p*< 0.01) with EY (0.554**) and BY (0.966**), while it exhibited negative and significant correlations with HI (-0.708**) and N concentration (-0.327**). The EY had positive and significant correlations with BY, CPY, macronutrient contents and uptake, and GY. The CPY was significantly and positively correlated with N (%), P (%), K (%), GNP, GPU, GKU and GY. In addition, grain N contents were positively and highly significantly correlated (*p*< 0.01) with P (%), K (%), GNP, GPU, GKU and GY. Similarly, grain P contents exhibited positive and significant correlations with K (%), nutrient uptake, and GY. There were positive correlations between grain K contents and GNU (0.426**), GPU (0.451**), GKU (0.697**) and GY (0.325**). The GNU was positively and strongly correlated (*p*< 0.01) with GPU (0.913**), GKU (0.882**) and GY (0.913**). The GKU had strong positive correlations (*p*< 0.01) with GY (0.900**). The GY exhibited positive and strong correlations (*p*< 0.01) with ED (0.810**), GN/E (0.636**), GW/E (0.940**), 100-grain weight (0.830**), EY (0.939**), BY (0.560**), HI (0.735**), CPY (0.913**), N (%) (0.298**), P (%) (0.314**), K (%) (0.325**) and GNU (0.913**).

**Table 5:** Correlations (Pearson correlation coefficient) between the study traits in maize as calculated from the combined data across two years.

Characters	ED	NG/E	GW/E	100-GW	SY	EY	BY	HI	CPY	N	P	K	GNU	GPU	GKU	GY
EL	0.168	0.261^*^	0.057	-0.08	0.264^*^	0.044	0.224^*^	-0.207	0.296^**^	0.507^**^	0.166	0.17	0.296^**^	0.136	0.163	0.114
ED		0.680^**^	0.821^**^	0.664^**^	0.507^**^	0.821^**^	0.657^**^	-0.007	0.812^**^	0.426^**^	0.201	0.427^**^	0.812^**^	0.759^**^	0.799^**^	0.810^**^
NG/E			0.643^**^	0.260^*^	0.585^**^	0.646^**^	0.665^**^	-0.198	0.636^**^	0.335^**^	0.393^**^	0.278^*^	0.636^**^	0.670^**^	0.598^**^	0.636^**^
GW/E				0.904^**^	0.564^**^	0.998^**^	0.757^**^	0.035	0.837^**^	0.231^*^	0.258^*^	0.288^**^	0.837^**^	0.886^**^	0.838^**^	0.940^**^
100-GW					0.376^**^	0.899^**^	0.577^**^	0.169	0.696^**^	0.101	0.132	0.233^*^	0.696^**^	0.748^**^	0.730^**^	0.830^**^
SY						0.554^**^	0.966^**^	-0.708^**^	-0.052	-0.327^**^	-0.108	-0.145	-0.052	0.078	0.029	0.129
EY							0.749^**^	0.041	0.833^**^	0.222^*^	0.257^*^	0.284^*^	0.833^**^	0.885^**^	0.836^**^	0.939^**^
BY								-0.551^**^	0.654^**^	0.481^**^	0.334^**^	0.331^**^	0.654^**^	0.580^**^	0.572^**^	0.560^**^
HI									0.777^**^	0.451^**^	0.345^**^	0.351^**^	0.777^**^	0.735^**^	0.713^**^	0.735^**^
CPY										0.656^**^	0.413^**^	0.426^**^	1.000^**^	0.913^**^	0.882^**^	0.913^**^
N											0.389^**^	0.394^**^	0.656^**^	0.375^**^	0.399^**^	0.298^**^
P												0.550^**^	0.413^**^	0.576^**^	0.480^**^	0.314^**^
K													0.426^**^	0.451^**^	0.697^**^	0.325^**^
GNU														0.913^**^	0.882^**^	0.913^**^
GPU															0.925^**^	0.955^**^
GKU																0.900^**^

*, ** Significant at P=0.05 and P= 0.01, respectively. EL is ear length (cm), ED is ear diameter (cm) and NG/E is number of grains/ear, GW/E is grain weight/ear (g), 100-GW is 100- grain weight (g), SY is stover yield (Mg ha^-1^), EY is ear yield (Mg ha^-1^) and BY is biological yield (Mg ha^-1^), HI is harvest index (%) and CPY is crude protein yield (kg ha^-1^), GNU is grain N uptake(kg ha^-1^), GPU is grain P uptake(kg ha^-1^), GKU is grain K uptake(kg ha^-1^)and GY is grain yield (Mg ha^-1^).

The direct and indirect effects of grain yield and the other yield components of maize across the two seasons are presented in **Table 6**. Grain weight/ear had positive and strong direct effects on grain yield (1.359), while the number of grains/ear and 100-grain weight exhibited negative effects (-0.144 and -0.361, respectively). For the indirect effects, only the number of grains/ear and 100-grain weight had positive effects on grain yield (0.874 and 1.229, respectively) through grain weight/ear.

**Table 6:** Direct (Diagonal) and indirect effect of yield components on maize grain yield across two years relative to correlation.

Characters	Number of grains/ear	Grain weight/ear (g)	100-grain weight (g)	Correlation with grain yield (Mg ha^-1^)
Number of grains/ear	-0.144	0.874	-0.094	0.636
Grain weight/ear (g)	-0.093	1.359	-0.327	0.940
100-grain weight (g)	-0.037	1.229	-0.361	0.830

## 4 Discussion

### 4.1 Response of maize yield attributes and crude protein yield to a reduced NPK strategy combined with biofertilizers and growth stimulants

Due to their vital roles in building plant tissues and all physiological processes, the decline in mineral N, P and K rates was accompanied by significant reductions in maize growth and ear parameters. Our results showed significant reductions in the ear parameters, including EL, ED, NG/E and GW/E, under partial replacement of mineral NPK fertilizers by biofertilizers. Replacing mineral fertilizers with biofertilizers has environmental importance by reducing the loss of chemical fertilizers to the environment but may have negative impacts on maize growth and yield (Gao et al., 2020). Higher reductions in GW/E were reported as compared with that in EL, ED and NG/E when using lower rates of NPK fertilizers, which indicates the importance of high rates of readily available NPK during grain filling (Zarabi et al., 2011). Biofertilizers are not direct sources of nutrient, but enhance the activity of soil microorganisms, which improves soil fertility by regulating the decomposition of organic matter, increasing nutrient solubility and protecting them against losses. This explains the reductions in ear parameters with the reduced NPK rates even when applying biofertilizers. We combined growth stimulants such as HA and AA to reduce the negative effect of reduced NPK rates on maize growth, and we found improvements in the maize ear parameters even under reduced NPK rates by 75%. The reductions in EL, ED, NG/E and GW/E were significantly affected by AA application, while the mixture of HA and AA with 75% NPK + biofertilizers increased those parameters over than applying 100% NPK. Under semiarid conditions, plants are subjected to drought periods during growth, which could reduce ear formation. In addition to containing N, P and K, the AA contains amino acids which enhance plant resistance to stress and reduce their effects on ear growth, grain formation and filling (Canellas et al., 2019). Additionally, HA contains organic substances and K, which promotes plant growth under stress conditions but does not contain high NPK like amino acids. Combining HA and AA exhibited superior effects on the ear parameters when compared with a single application of HA or AA. For cleaner maize production, we suggest combining lower rates of chemical NPK fertilizers with biofertilizers and HA and AA mixtures. On the other hand, GY, SY, EY, BY, HI and CPY recorded significant variations in their responses to the combined application of chemical NPK fertilizers, biofertilization and growth stimulants (HA and AA). Sharp reductions in these attributes were exhibited by reducing the mineral NPK rate by 25-100%, even with biofertilization. Similarly, increases in grain and stover yields with increasing N, P and K rates were reported (Gul et al., 2015). Higher N, P and K uptakes by maize plants produce higher LAIs, which activate photosynthesis and lead to greater dry matter production in terms of grain and stover yields (Canellas et al., 2019). Applying biofertilizers did not noticeably compensate for the sharp reductions in yield attributes that resulted from the reduced mineral fertilizer rate, which indicates less efficient of biofertilization under low NPK rates. Only an improvement by 12.5% in maize yields under biofertilization was reported by the meta-analysis study of Schmidt and Gaudin (2018). They found that biofertilizers were more effective under controlled conditions than under open field conditions, as field conditions might not be appropriate for microorganism activity, especially under semiarid conditions. Applying HA decreased the adverse effect of lower rates of mineral fertilization but not as much as AA or the mixture of HA and AA, because HA could only promote plant resistance to environmental stresses through its organic components. AA had the same effect as the HA and AA mixture on improving the yield attributes to exhibit higher GY, SY, EY and BY than by applying 100% NPK only. Similarly, there were increases in grain yields and yield attributes, as well as grain protein contents and GNU, with the application of HA or AA (Khan et al., 2019). In addition, AA is a direct source of N, P and K, which promotes plant resistance to stress under arid conditions (drought) and increases protein formation, photosynthesis and grain formation and filling (Szczepaniak et al., 2018). It is worth mentioning that HI recorded a contradictory response, for which the highest HI was reported when applying 25% NPK + biofertilizers, which means higher grain formation against dry matter. The HI measures the relative investment of plant resources in their reproductive parts (Unkovich, 2010). The CPY increased with increasing the replacement of NPK fertilizer with biofertilizers combined with foliar application of HA and AA. This response could result from enhanced soil fertility with high organic matter and N contents, which increased grain yields (White, 2009; El-Sobky, 2016), amino acid formation (Jiang et al., 2019) and mineralization of soil organic N (Li et al., 2003), and accelerated the physiological and biochemical processes of the plants (Rawal and Kuligod, 2014). That increased the N concentration and N uptake. In addition, humic acid and amino acids enhance plant functions such as photosynthesis, protein synthesis, phytohormone activation, total amino acids and grain contents of N, P and K (Ragheb, 2016; Canellas et al., 2019; Khan et al., 2019).

### 4.2 Effect of reduced NPK rates combined with biofertilizers and growth stimulants on maize macronutrient contents and uptake

Nutrient contents and uptakes by maize grains have a strong positive correlation with mineral fertilization rates (Luan et al., 2020; Li et al., 2021). Significant reductions in N, P and K contents were exhibited under chemical NPK rates that were lower than 50% even combined with biofertilizers. Meanwhile, the nutrient uptakes and crude protein yields recorded sharp reductions with decreasing NPK rate of less than 100% with biofertilizers. These results were correlated with the previous sharp reductions in grain yield, which demonstrated the role of biofertilizers for continuous, but not rapid or high supply with NPK like chemical fertilizers to maximize yield (Sarajuoghi et al., 2013; Kubheka et al., 2020a). We applied a biofertilizer mixture of N_2_-fixing bacteria and P- and K-solubilizing bacteria, which increased soil macronutrient availability and uptake by plants (Goebel et al., 2016). The biofertilizers produced a compound that could be synthesized by bacteria or facilitate nutrient uptake from the environment. The application of growth stimulants, especially AA, under 75% NPK + biofertilizers, caused significant increases in the N and P contents and their uptakes to have the same values like that of 100% NPK. On the other hand, the K contents and uptakes recorded their highest values when applying 50% NPK + biofertilizers with HA. These results demonstrate the role of AA-containing amino acids and N and P nutrients in improving the assimilation of these nutrients in grains, which also proves the stronger effect of AA on grain yield compared with other stimulants (Hegab et al., 2020). There were increments in grain N concentrations and total N uptakes of maize with N fertilizer applications (Niaz et al., 2016). The HA is a source of organic acids and K, which could prevent sharp reductions in grain yield under environmental stress and can significantly supply plants with K only, which is consistent with our results. Increased K contents with the reduction of NPK rate by 50% refer to the antagonistic effect of high N rates on K uptake by maize grains. There are no previous studies on the combined effect of HA and/or AA on the N, P and K contents and uptakes by maize grains; however, there were increments in grain N and P contents by 21.3 and 15.2%, respectively, under AA application when compared with HA (Hegab et al., 2020). The K contents increased by 22.7% under HA application compared with AA application.

### 4.3 Correlations and path coefficients among grain yields and yield attributes and macronutrient contents and uptake

The correlations among the examined traits may be due to the consequence of the genetic associations among the studied parameters. The correlation and path analysis (**Table 5**) revealed that grain yield had significant relationships with the yield components, macronutrient content and nutrient uptake. These findings suggested that the improvement in maize grain yields is linked to an increase in those traits that might have positive impacts on grain yield. Similarly, significant positive correlations among maize grain yields and yield attributes as well as with grain quality were reported [Ali (2016); Reddy and Jabeen (2016)]. The results revealed that grain weight/ear was considered to be the major yield component that maize breeders should consider to produce high-yielding maize. Similar results have been reported by several investigators (Nataraj et al., 2014; Ali, 2016; Reddy and Jabeen, 2016).

## 5 Conclusions

The efforts to obtain cleaner production are continuously increasing due to the environmental hazards that are caused by the intensive application of chemical fertilizers, especially N, P and K. Although the replacement of these chemicals with biofertilizers is a strongly recommended strategy, numerous findings have indicated that such replacements are an inefficient economic strategy. As shown by our study, there were sharp reductions in the maize yield attributes when replacing chemical NPK fertilizer by 25% to 100% with biofertilizers. For example, the grain yield was halved when reducing the recommendation rate of NPK fertilizers by 25%. Bio-stimulants, including humic (HA) and amino acids (AA), act against these reductions and significantly improved the maize yield quantities and qualities under 75% NPK more than for the recommended NPK rate. Moreover, the best yield attributes were obtained under the application of 75% NPK with HA and AA as compared with 100% of NPK fertilizers. Generally, the mixture of HA and AA reported the greatest effects, which was followed by AA and then HA. We strongly recommend combining the reduced amounts of chemical fertilizers with biological fertilizer, and HA and AA as strategies to obtain optimal maize yields and quality under semiarid conditions with less environmental hazards.

## Data availability statement

The original contributions presented in the study are included in the article/**Supplementary Material**. Further inquiries can be directed to the corresponding author.

## Author contributions

AA: Investigation, methodology, data curation, writing - original draft. E-SE-S: Data curation, resources, investigation, methodology, validation, writing - original draft. JZ: Writing – review & editing, funding acquisition. All authors contributed to the article and approved the submitted version.

## Acknowledgments

The present study was supported by Guangdong Provincial Special Project of Rural Revitalization Strategy (Document No. [2021] 12), Science and Technology Planning Project of Guangdong Province of China (grant number 2019B030301007) and Guangdong Laboratory for Lingnan Modern Agriculture (NT2021010).

## Conflict of interest

The authors declare that the research was conducted in the absence of any commercial or financial relationships that could be construed as a potential conflict of interest.

## Publisher’s note

All claims expressed in this article are solely those of the authors and do not necessarily represent those of their affiliated organizations, or those of the publisher, the editors and the reviewers. Any product that may be evaluated in this article, or claim that may be made by its manufacturer, is not guaranteed or endorsed by the publisher.
