# Supplementary material for: Optimizing maize yields using growth stimulants under the strategy of replacing chemicals with biological fertilizers
Source: Front Plant Sci. 2022 Nov 25;13:1069624. doi: 10.3389/fpls.2022.1069624 (PMC9732421; doi:10.3389/fpls.2022.1069624)
Supplement: Supplementary file 1 [file Table_1.docx]

**Table S1.** Monthly average minimum (Min) and maximum (Max) temperatures, relative humidity (RH), and rainfall in 2019 and 2020 growing seasons.

| Month | Min (° C) | Max (° C) | | RH (%) | | Rainfall (mm) | |
| --- | --- | --- | --- | --- | --- | --- | --- |
|  | First season (2019) | | | | | | |
| May | 17.17 | | 34.61 | | 38.12 | | 0.00 |
| June | 20.11 | | 35.33 | | 49.66 | | 0.00 |
| July | 24.23 | | 35.97 | | 52.79 | | 0.00 |
| August | 22.77 | | 36.01 | | 53.96 | | 0.00 |
| September | 23.91 | | 33.00 | | 57.91 | | 0.00 |
|  | Second season (2020) | | | | | | |
| May | 18.44 | | 32.19 | | 42.69 | | 0.00 |
| June | 20.78 | | 34.22 | | 44.46 | | 0.00 |
| July | 24.14 | | 34.69 | | 55.84 | | 0.00 |
| August | 22.01 | | 35.47 | | 55.73 | | 0.00 |
| September | 20.33 | | 35.22 | | 59.62 | | 0.00 |
